# Tandem Mass-Remainder Analysis of Industrially Important Polyether Polyols

**DOI:** 10.3390/polym12122768

**Published:** 2020-11-24

**Authors:** Mahir Hashimov, Ákos Kuki, Tibor Nagy, Miklós Zsuga, Sándor Kéki

**Affiliations:** 1 Department of Applied Chemistry, Faculty of Science and Technology, University of Debrecen, Egyetem tér 1, H-4032 Debrecen, Hungary; mahir.hashimov@science.unideb.hu (M.H.); kuki.akos@science.unideb.hu (Á.K.); nagy.tibor@science.unideb.hu (T.N.); zsuga.miklos@science.unideb.hu (M.Z.); 2 Doctoral School of Chemistry, University of Debrecen, Egyetem tér 1, H-4032 Debrecen, Hungary

**Keywords:** tandem mass spectrometry, Mass-remainder analysis, polyurethanes, polyols

## Abstract

The characteristics of the polyalkylene oxide polyether polyols highly influence the properties of final polyurethane products. As a novel approach, in order to gain structural information, the recently invented data mining procedures, namely the Mass-remainder analysis (MARA) and the Multistep Mass-remainder analysis (M-MARA) are successfully applied for the processing of tandem mass spectrometry (MS/MS) data of various industrially important polyether polyols. M-MARA yields an ultra-simplified graphical representation of the MS/MS spectra and sorts the product ions based on their double bond equivalent (DBE) values. The maximum DBE values unambiguously differentiate among the various polyether polyols. Accordingly, the characteristic DBE values were 0, 1 for the linear diol polyethers, 0, 1, 2 for the three-arm, and 0, 1 2, 3, 4 for the six-arm polyether polyols. In addition, it was also found that the characteristic collision energy necessary for the optimum fragmentation yield depended linearly on the molecular weight of the polyols. This relationship offers an easy way for instrument tuning to gain structural information.

## 1. Introduction

Owing to their very diverse chemical structures and superior properties, polyurethanes (PUs) have become one of the most important classes of industrial polymers. Hence, PUs are used in a wide variety of applications such as flexible and rigid foams, adhesives, coatings, sealants, elastomers, fiber composite materials, paints and so on [1,2]. Furthermore, PUs are also extensively used in the automotive industry. In addition to their traditional automotive applications e.g., cushioning, bumper or sound insulation materials, PUs find their utilities as novel structural components and alternative adhesives [3,4]. Polyurethanes are synthetized by the reaction of di- or polyisocyanates with polyols or mixture of various polyols. One of the reasons of the divers PUs applications stems from the versatile chemistry of polyols. Polyalkylene oxide polyether polyols, which are the subjects of our study, are the most important group of polyols for manufacturing PUs [1]. The key characteristics, such as the number of reactive hydroxyl end-groups and molecular structure greatly influence the properties of the final PU product. Therefore, the structural characterization of the polyols is essential not only for the development of novel polyurethane materials but for the quality control of PU products [5]. Mass spectrometry combined with soft ionization methods including matrix-assisted laser desorption/ionization (MALDI) [6,7] or electrospray ionization (ESI) mass spectrometry [8] can be effectively used to analyze polyether polyols. In addition to a single MS, tandem mass spectrometry (MS/MS) can provide additional information required for complete structural characterization of the given polymer components [9]. Linear diol polyethers, particularly polyethylene glycols (PEG) and polypropylene glycols (PPG) are extensively studied by single and tandem mass spectrometry and their characterizations using soft ionization methods are well documented [10,11,12,13,14,15,16,17,18,19,20]. However, the mass spectrometric analysis of the multi-arm (star) polyol polymers, copolymers, or polymer/copolymer blends is a challenging task and it may suffer from several drawbacks such as the presence of isobar and isomer compounds, overlapping peaks and structural diversity. Such typical analytical difficulties arising from the presence of isomer/isobar compositions are demonstrated in Table 1 that lists some isomer/isobar molecular species for five industrially important polyether polyols. The chemical structures of different types of polyether polyols are shown in the Supporting Information (Figure S1).

The unambiguous structural elucidation of these complex polymer systems including multi arm (star) polyether polymers or copolymers runs into difficulties even when using sophisticated MS/MS techniques as demonstrated in a few reports [15,21,22]. Recently, we have provided a simple algorithm, called Mass-remainder analysis (MARA) and its improved method, the Multistep Mass-remainder analysis (M-MARA) for the processing and assessment of complex mass spectra [23,24,25,26]. In this work, we implement M-MARA for the evaluation of the tandem mass spectra of industrially important polyether polyols having various chemical structures.

## 2. Materials and Methods

### 2.1. Chemicals

The Rokopol RF551, RF4855 and G1000 polymers were purchased from PCC Group (Brzeg Dolny, Poland). PE3100, PEG600 and PPG1000 polymers were received from BASF (Ludwigshafen, Germany), Merck (Darmstadt, Germany), and Sigma Aldrich (Steinheim, Germany), respectively. 

Methanol (HPLC-MS grade) was obtained from VWR International (Leuven, Belgium). All polymers were dissolved in methanol at a concentration of 0.01 mg/mL. Lithium chloride (Sigma Aldrich, Steinheim, Germany) was used as an ionizing agent (4 mg/mL). 10 μL of ionizing agent was added to each 1 mL-polymer samples, then injected into the instrument.

The investigated samples are listed in Table 2. 

### 2.2. Electrospray Quadrupole Time-of-Flight Mass Spectrometry (ESI-QTOF MS)

A Maxis II type Qq-TOF MS instrument (Bruker Daltonik, Bremen, Germany) equipped with an Apollo II electrospray ion-source was used in positive ion mode. The spray voltage was 4.0 kV. The resolution of the instrument was 40 000 at *m*/*z* 400 (FWHM), and the mass accuracy was better than 1 ppm (external calibration). N_2_ was utilized as drying gas (200 °C, 4.0 L/min), nebulizer gas (0.5 bar) and collision gas. The collision energy was varied in the range of 50–130 eV in the laboratory frame. All precursor ions were selected with an isolation width of *m*/*z* 2.00. The mass spectra were recorded with a digitizer at a sampling rate of 2 GHz. The spectra were calibrated externally by ESI tune mix provided by Bruker Daltonik (Bremen, Germany). The spectra were evaluated with Compass Data Analysis 4.4 software (Bruker, Bremen, Germany). The sample solutions were introduced directly into the ESI source with a syringe pump (Cole-Parmer Ins. Co., Vernon Hills, IL, USA) at a flow rate of 3 μL/min.

## 3. Results and Discussion

**Figure 1:**
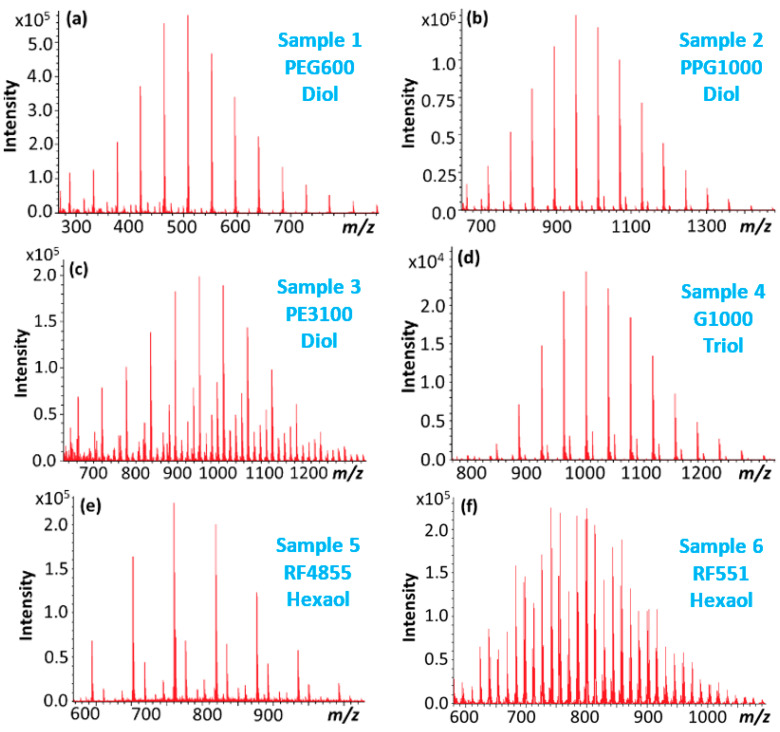
ESI-MS spectra ([M + Li]^+^) of (**a**) polyethylene glycol, Sample 1. (**b**) polypropylene glycol, Sample 2. (**c**) polyethylene glycol/polypropylene glycol copolymer, Sample 3. (**d**) glycerol-based polypropylene glycol, Sample 4. (**e**) sorbitol-based polypropylene glycol, Sample 5. (**f**) sorbitol-based polyethylene glycol/polypropylene glycol, Sample 6.

Six different polyether polyols of various structures were subjected to MS/MS investigations as shown in Table 2. Since the MS/MS of the lithium-attached ([M + Li]^+^) ions of the polyethers have been reported to give rich product ion spectra suitable for structural elucidation [10,11,12,13,14,15] we used LiCl salt as the cationization reagent for enhancing the formation of [M + Li]^+^ ions primarily. Indeed, the ESI-MS spectra of the polyether polyols are dominated by the lithium-attachment ions (Figure 1). 

**Figure 2:**
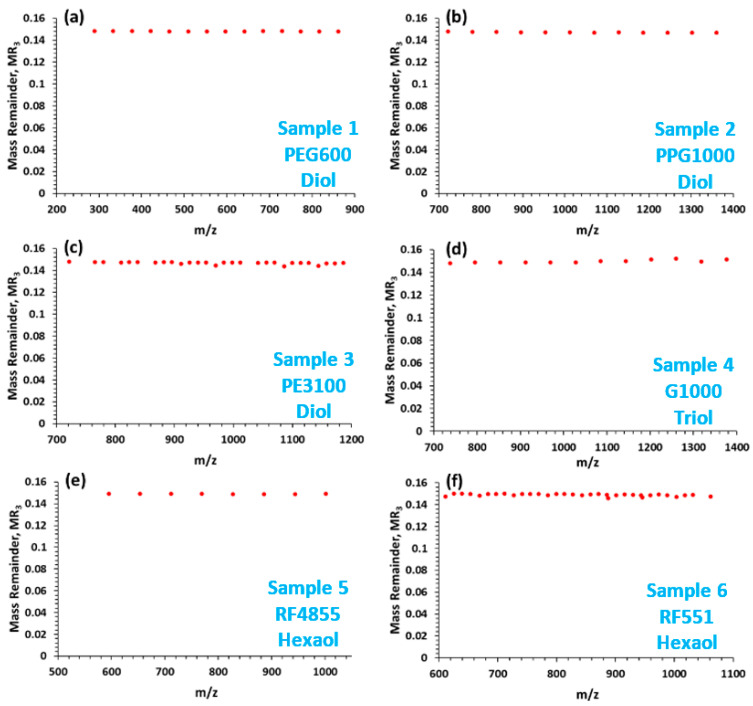
MR_3_ versus *m*/*z* plots constructed from the ESI-MS spectra (with lithium-attached ions) of (**a**) polyethylene glycol, Sample 1. (**b**) polypropylene glycol, Sample 2. (**c**) polyethylene glycol/polypropylene glycol copolymer, Sample 3. (**d**) glycerol-based polypropylene glycol, Sample 4. (**e**) sorbitol-based polypropylene glycol, Sample 5. (**f**) sorbitol-based polyethylene glycol/polypropylene glycol, Sample 6.

As detailed in our previous work^25^, Multistep Mass-remainder analysis (M-MARA) performs sequential calculations of the remainders, that eliminates the homologous connections present in a complex system. Therefore, M-MARA provides highly simplified plots. Equation (1) reveals the general formula for the three-step M-MARA:

MR_3_ = [(*m*/*z* MOD R_1_) MOD R_2_] MOD R_3_ (1)

where the modulo (MOD) operation finds the mass remainder (MR_n_) after the division by R_n_ (n = 1, 2, 3). In order to eliminate the periodicities generated by the repeat units EO (i.e., C_2_H_4_O = 44.02622 Da) and PO (i.e., C_3_H_6_O = 58.04187 Da), R_1_ = 58.04187, R_2_ = 1.979265, and R_3_ = 0.160795 were chosen for the MARA base units corresponding to the masses of PO, 4×EO-3×PO, and 22 × PO-29 × EO, respectively. (The reason behind the selection of the base units is detailed in the next paragraph.) The results of the calculations by M-MARA, i.e., MR_3_ versus *m*/*z* plots of the ESI-MS spectra of the six polyethers are shown in the Supporting Information (Figure S2). Furthermore, the target lithium-attached ions can be filtered easily by means of M-MARA, thus even more simplified plots can be constructed as demonstrated in Figure 2. As seen in Figure 2, the M-MARA plots of all the polyether polyols listed in Table 2 show only a single horizontal MR-line at MR_3_ = 0.149. In addition, it is also valid even for the four-arm pentaerythritol base PEG/PPG copolymers (see Table 1). Moreover, if the initiator moiety is ethylenediamine (see Table 1, line 5) the MR-line of the lithiated peaks is at MR_3_ = 0.161. Hence, M-MARA is capable of making a difference between the glycerin/sorbitol/pentaerythritol base propoxylated polyols or their blends and the ethylenediamine base propoxylated polyols. 

The fundamental of M-MARA is the selection of suitable base units (R_1_, R_2_, R_3_ in Equation (1)). The aim of the multiple steps is to achieve efficient sorting to evaluate the complex tandem mass spectra and highlight the hidden composition or structural differences. Calculating MR_1_ in the first step (see Equation (1)), mass remainder series are generated and the chemical composition of the species can be determined by the reference values as we have shown previously [23]. However, the MR_1_ versus *m*/*z* plot for unknown and/or complex samples, e.g., for copolymers and/or their blends may be rather fuzzy making the interpretation of the plot very difficult. (In the case of copolymers the difficulties on the interpretation come from the composition differences obtained by multiple replacements of the monomer units). Although visual identification of these characteristic differences is possible, however, these values can easily be read out from the series of MR values. After the first step (using either of the monomer units as the base unit), the second base unit can be determined from the series of MR_1_ values. If two neighboring MR_1_ values arranged according to their *m*/*z* values are not the same, the difference between them can be used as the base unit for the next step of MARA. For example, in the case of EO-PO copolymer systems, where the first step base unit is PO, the MR_1_ versus *m*/*z* plot contains multiple series of horizontal MR-lines with characteristic differences among them. These differences are originated from the replacement of EO/PO units, namely the 14.015650, 1.979265 and 0.160798 differences correspond to the replacements of PO-EO, 4EO-3PO and 22PO-29EO, respectively. Similar differences are expected in the tandem mass spectrum of these copolymers. Performing the three steps of M-MARA the periodicities generated by the EO/PO replacements can be eliminated, the MR_3_ versus *m*/*z* plot of the MS spectrum is a single MR-line, as seen in Figure 2. However, the polymer/copolymer fragmentation yields other differences and thus additional MR-lines occur in the MR_3_ versus *m*/*z* plot of the MS/MS spectrum. In the case of EO-PO copolymers, the different MR-lines have different DBE (double bond equivalent) values, formed during the fragmentation, as will be discussed in the next section. 

**Figure 3:**
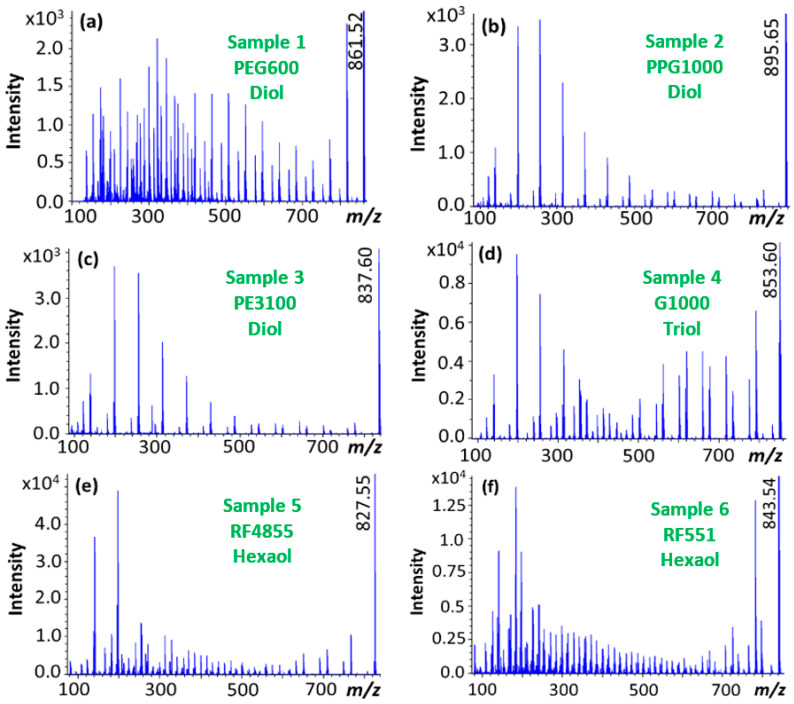
ESI-MS/MS spectra ([M + Li]^+^) recorded at 90 eV laboratory frame collision energy of (**a**) polyethylene glycol, Sample 1. (**b**) polypropylene glycol, Sample 2. (**c**) polyethylene glycol/polypropylene glycol copolymer, Sample 3. (**d**) glycerol-based polypropylene glycol, Sample 4. (**e**) sorbitol-based polypropylene glycol, Sample 5. (**f**) sorbitol-based polyethylene glycol/polypropylene glycol, Sample 6.

In the next step, tandem mass spectrometry experiments were performed in order to make distinction between the diol, triol and hexaol polyether polymers/copolymers by exploring their molecular structure. Figure 3 shows the MS/MS spectra of lithiated precursor ions ([M + Li]^+^) with approximately the same mass-to-charge ratio for each sample (Samples 1–6). 

**Figure 4:**
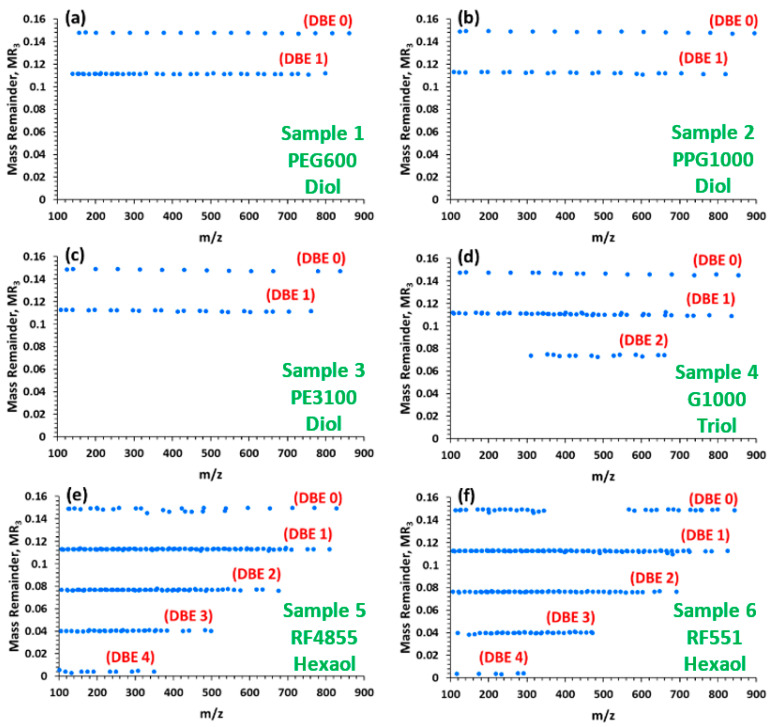
MR_3_ versus *m*/*z* plots of the MS/MS spectra depicted in Figure 3 of (**a**) polyethylene glycol, Sample 1. (**b**) polypropylene glycol, Sample 2. (**c**) polyethylene glycol/polypropylene glycol copolymer, Sample 3. (**d**) glycerol-based polypropylene glycol, Sample 4. (**e**) sorbitol-based polypropylene glycol, Sample 5. (**f**) sorbitol-based polyethylene glycol/polypropylene glycol, Sample 6.

As seen in Figure 3 (especially in Figure 3d–f), the tandem mass spectra of the propoxylated polyols inherit the complexity of their single MS spectra (Figure 1). Therefore, manual assignment of the individual peaks is particularly challenging and time-consuming. Let us examine whether M-MARA can simplify these peak-rich spectra and, even more importantly, can reveal the structural differences between the diol, triol and hexaol polyether polyols. Figure 4 shows the results of the Multistep Mass-remainder analysis, i.e., the MR_3_ versus *m*/*z* plots of the MS/MS spectra of the six-arm polyether polyols. 

As we have seen in Figure 2, the M-MARA plots of Sample 1–6 are identical, they contain merely a single MR-line. On the contrary, if the M-MARA plots are constructed from the corresponding tandem mass spectra (Figure 4), the structural differences occur markedly, namely the increase of the number of MR-lines indicates an increase in the number or arms of the multi-arm polyether polyol structures. In Figure 4, the top MR-line at MR_3_ = 0.149 is the same as in Figure 2, which corresponds to the A product ion series formed by losses of monomeric (EO or PO) units, using the notations originally proposed by Lattimer [10]. The second horizontal MR-line from the top is associated with the fragmentation pathways yielding the B (vinyl-terminated) and C (formyl-terminated) fragment ion series. Table 3 lists the possible product ion compositions and their MR_3_ values of the diol-type PEG/PPG polymers or copolymers. 

The B and C fragments are formed by H_2_ elimination resulting DBE = 1, where DBE stands for double bond equivalent corresponding, in our case, to the number of double bonds. As seen in Table 3, MR_3_ = 0.036 of H_2_, consequently, the MR_3_ values of the B and C series equal 0.149 − 0.036 = 0.113 (see Figure 4). The additional horizontal MR-lines in the MR_3_ versus *m*/*z* plots of the three-arm and six-arm polyethers can be associated to DBE = n, at MR_3_ = 0.149 − n × 0.036, where n = 2, 3, 4 (see Figure 4d–f). It is likely that the bonds between the arms and the glycerin or sorbitol base are fragile compared to the bonds in the linear diol polyethers, therefore, multiple collisions between the fragment ions and the collision gas result in further decomposition yielding fragment ions via additional H_2_ eliminations. Furthermore, theoretically the multiple fragmentation can yield maximum DBE = 2 for the linear diol polyethers (both chain ends are vinyl or formyl terminated), DBE = 3 and 6 for the three- and six-arm polyether polyols, respectively (all the arms are vinyl or formyl terminated). It should be noted, that the width of the isolation window of the precursor ion selection may affect the fragmentation pattern. However, in our experiments multiple precursor ion selection are only due to EO/PO replacements, that does not modify the MR_3_ plots.

In order to observe informative fragmentation in the collision-induced dissociation (CID) analysis of the polyether polyols, the collision voltage was adjusted to the characteristic collision voltage corresponding to SY = 0.20 (CCV^20%^), where SY stands for survival yield that is a quantitative measure to describe the efficiency of fragmentation and calculated as the ratio of the precursor ion intensity to the sum of all fragment ion plus precursor ion intensities [27]. In preliminary experiments, the molecular mass dependence of CCV^20%^ (as well as that of CCV^50%^, the collision voltage necessary to obtain 50% fragmentation) was studied for the polyether polyols. 

**Figure 5:**
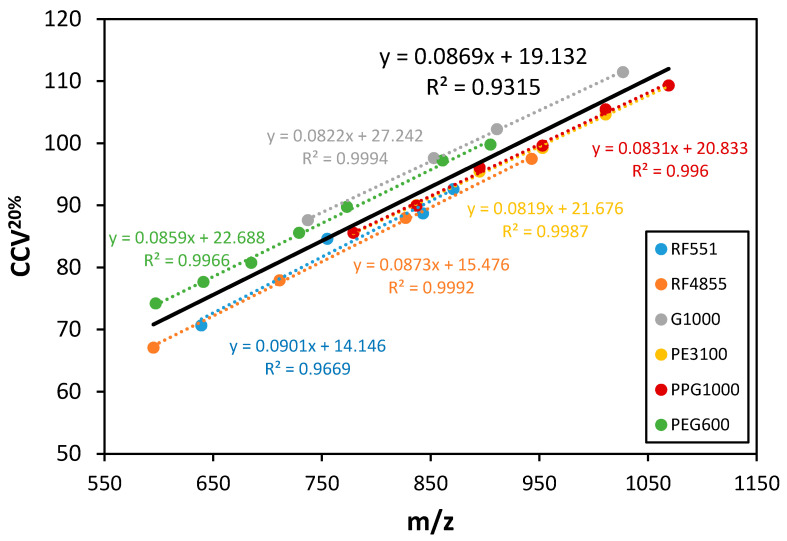
Characteristic collision voltage (CCV^20%^) versus *m*/*z* plots of the six polyether polyols. The average (black line) is the trend line taking into account all the data points.

Linear relationship was found between the CCV^20%^ values and the mass-to-charge ratio for each sample as shown in Figure 5. (CCV^50%^ versus *m*/*z* plots are depicted in Supporting Information Figure S3). Moreover, the graph lines are parallel and locate close together. It suggests, that the characteristic collision voltage (e.g., CCV^20%^) only slightly depends on the initiator moiety. These results propose an easy method for instrument tuning to gain structural information, specifically to differentiate among the polyether polyols based on the DBE lines appearing in their M-MARA plots (Figure 4). Taking an approximate average of the CCV^20%^ versus *m*/*z* lines a calibration curve can be constructed (see Figure 5, black line). This calibration curve is used to determine the CCV^20%^ value (say the collision voltage that results SY = 0.20) for a given precursor ion mass. By setting this collision voltage (CCV^20%^) the DBE lines appear in the MR_3_ versus *m*/*z* plots, and the DBE values (or the number of DBE lines) unambiguously differentiate among the various polyether polyols. The characteristic DBE values were 0-1 for the linear diol polyethers (2 DBE lines), 0–2 for the three-arm (3 DBE lines), and 0-4 for the six-arm polyether polyols (5 DBE lines), as seen in Figure 4.

## 4. Conclusions

The complex, peak-rich MS/MS spectra of various polyether polyols were successfully analyzed by the Multistep Mass-remainder analysis (M-MARA), our recently invented data mining procedure. By M-MARA, the graphical representation of the tandem mass spectra can drastically be simplified. The sequential calculation of the mass remainders eliminates the periodicities present in the spectra, yielding a diagram in which the dots representing the product ions are aligned in horizontal lines according to their DBE values. The theoretical maximum of the DBE values equals to the arms of the polyether polyol: it is 2 for the linear (quasi 2-arm) polyols, 3 and 6 for the glycerin and sorbitol base polyols, respectively. As the increase in the DBE values (i.e., the increase of the number of DBE lines in the M-MARA plots) is due to multiple fragmentation, the actually appearing DBE maximum depends on the applied collision voltage/energy. We found, that using the characteristic collision voltage CCV^20%^ for the CID experiments, the maximum DBE value was 1, 2 and 4 for the linear, glycerol base, and sorbitol base polyether polyols, respectively.

Due to the complexity of the MS and MS/MS spectra of copolymers containing thousands of mass peaks, the data processing and visualization methods have great relevance. The detailed structural analysis of copolymers performed by our novel method is crucial to understand the physical and chemical behavior of copolymers. Beyond the analysis and differentiation of polyether polyols by the identification of DBE values of the fragments, M-MARA is suitable to highlight other type of distinctions such as number of heteroatoms, rings or aromatic moieties.

## Figures and Tables

**Table 1:** Typical examples of isomer/isobar molecular species of diol, triol (glycerin base), tetraol (pentaerythritol base), tetraol (ethylenediamine base), and hexaol (sorbitol base) polyethylene glycol (PEG)/ polypropylene glycol (PPG) copolymers.

No.	Type	Structure	Elemental Composition	Monoisotopic Mass (Da)
1	Diol	H_2_O(C_2_H_4_O)_9_(C_3_H_6_O)_3_	C_27_H_56_O_13_	588.3721
2	Triol	C_3_H_8_O_3_(C_2_H_4_O)_6_(C_3_H_6_O)_4_	C_27_H_56_O_13_	588.3721
3	Tetraol	C_5_H_12_O_4_(C_2_H_4_O)_5_(C_3_H_6_O)_4_	C_27_H_56_O_13_	588.3721
4	Hexaol	C_6_H_14_O_6_(C_2_H_4_O)_0_(C_3_H_6_O)_7_	C_27_H_56_O_13_	588.3721
5	Tetraol	(NH_2_)_2_C_2_H_4_(C_2_H_4_O)_12_(C_3_H_6_O)_0_	C_26_H_56_N_2_O_12_	588.3833

**Table 2:** Composition, structure, and the number average molecular weight (M_n_) of the investigated polyether polyols. Where EO and PO stand for ethylene oxide and propylene oxide, respectively.

	Name	Composition	Base	Structure	M_n_ (MS) (g/mol)
Sample 1	PEG600	EO		Diol—linear	600
Sample 2	PPG1000	PO		Diol—linear	1000
Sample 3	PE3100	EO/PO		Diol—linear	1000
Sample 4	G1000	PO	glycerol	Triol—3 arms	1000
Sample 5	RF4855	PO	sorbitol	Hexaol—6 arms	600
Sample 6	RF551	EO/PO	sorbitol	Hexaol—6 arms	800

**Table 3:** Product ion series of the diol PEG/PPG copolymers and their MR_3_ values.

Series	Formula	Chain Termination	MR_3_
A	Li[HO(C_2_H_4_O)_n_(C_3_H_6_O)_m_H]^+^	hydroxyl	0.149
B	Li[HO(C_2_H_4_O)_n_(C_3_H_6_O)_m_CHCH_2_]^+^	vinyl	0.113
B	Li[HO(C_2_H_4_O)_n_(C_3_H_6_O)_m_CHCHCH_3_]^+^	vinyl	0.113
C	Li[HO(C_2_H_4_O)_n_(C_3_H_6_O)_m_CH_2_CHO]^+^	formyl	0.113
C	Li[HO(C_2_H_4_O)_n_(C_3_H_6_O)_m_CHCH_3_CHO]^+^	formyl	0.113
	H_2_		0.036

## Supplementary Materials

The following are available online at https://www.mdpi.com/2073-4360/12/12/2768/s1. Figure S1: Structures of different types of polyether polyols, Figure S2: MR3 vs *m*/*z* plots constructed from the MS spectra of (a) polyethylene glycol, Sample 1. (b) polypropylene glycol, Sample 2. (c) polyethylene glycol / polypropylene glycol copolymer, Sample 3. (d) glycerol based polypropylene glycol, Sample 4. (e) sorbitol based polypropylene glycol, Sample 5. (f) sorbitol based polyethylene glycol / polypropylene glycol, Sample 6, Figure S3. CCV50% vs *m*/*z* plots of the six polyether polyols.
