# Supplementary material for: Tandem Mass-Remainder Analysis of Industrially Important Polyether Polyols
Source: Polymers (Basel). 2020 Nov 24;12(12):2768. doi: 10.3390/polym12122768 (PMC7761062; doi:10.3390/polym12122768)
Supplement: Supplementary file 1 [file polymers-12-02768-s001.pdf]

polymers

Supporting Information of

Tandem Mass-Remainder Analysis of Industrially Important Polyether Polyols

Mahir Hashimov [1,2], Ákos Kuki [1], Tibor Nagy [1], Miklós Zsuga [1] and Sándor Kéki [1,*]

[1] Department of Applied Chemistry, Faculty of Science and Technology, University of Debrecen, H-4032 Debrecen, Egyetem tér 1, Hungary
[2] University of Debrecen, Doctoral School of Chemistry, H-4032 Debrecen, Egyetem tér 1, Hungary
* Correspondence: keki.sandor@science.unideb.hu

Received: 2 November 2020; Accepted: 22 November 2020; Published: date

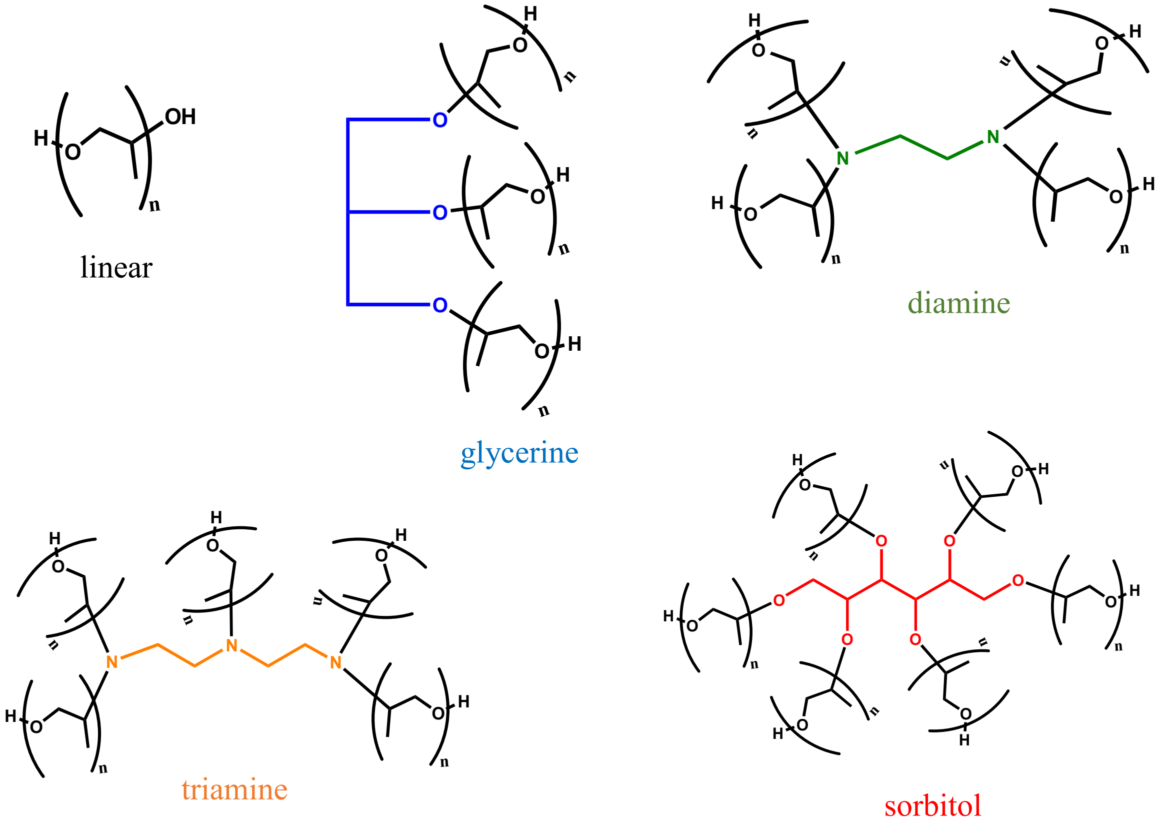

Figure S1. Structures of different types of polyether polyols.

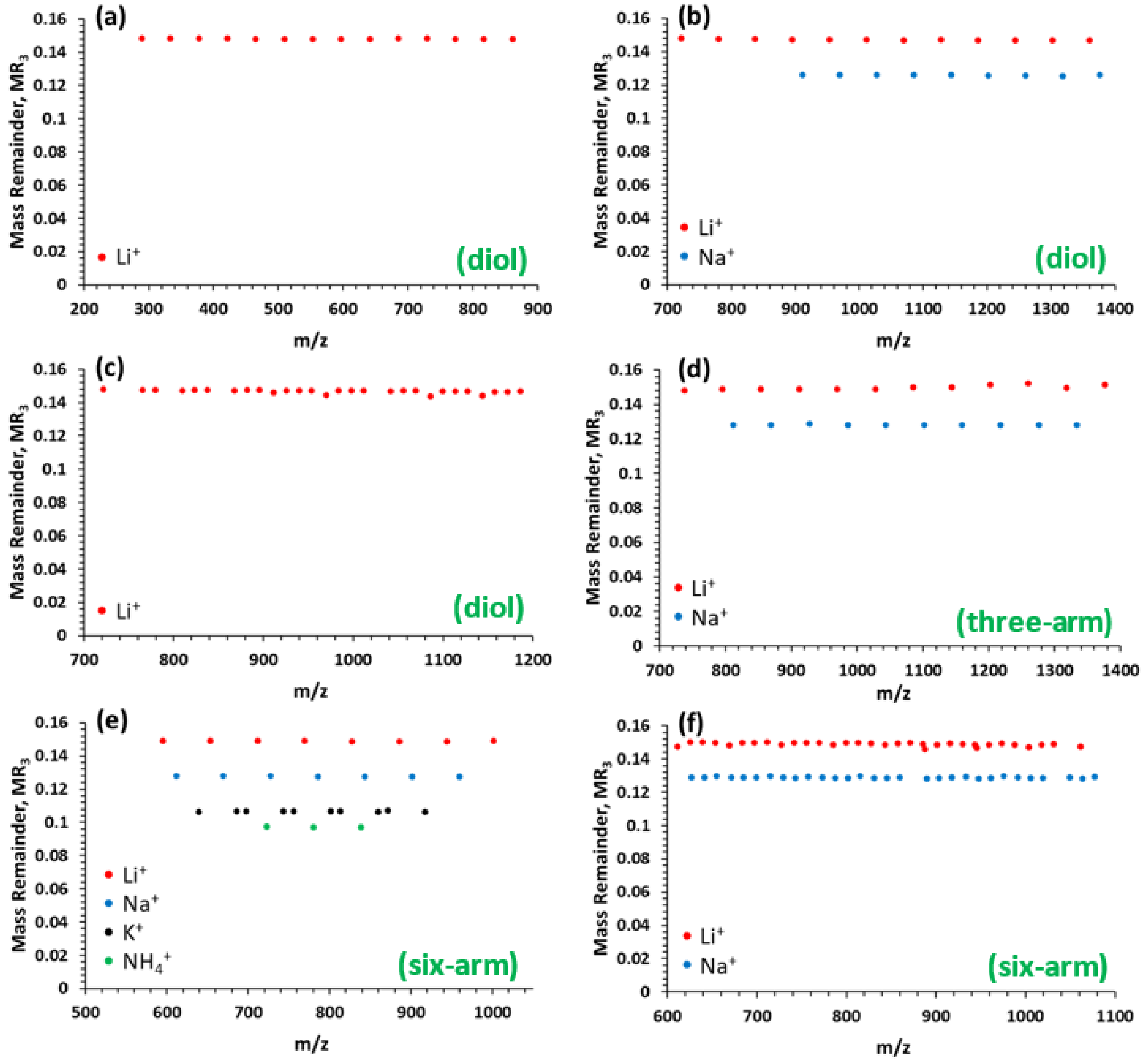

Figure S2. MR3 versus *m*/*z* plots constructed from the MS spectra of (**a**) Polyethylene glycol, Sample 1. (**b**) Polypropylene glycol, Sample 2. (**c**) Polyethylene glycol / polypropylene glycol copolymer, Sample 3. (**d**) Glycerol based polypropylene glycol, Sample 4. (**e**) Sorbitol based polypropylene glycol, Sample 5. (**f**) Sorbitol based polyethylene glycol / polypropylene glycol, Sample 6.

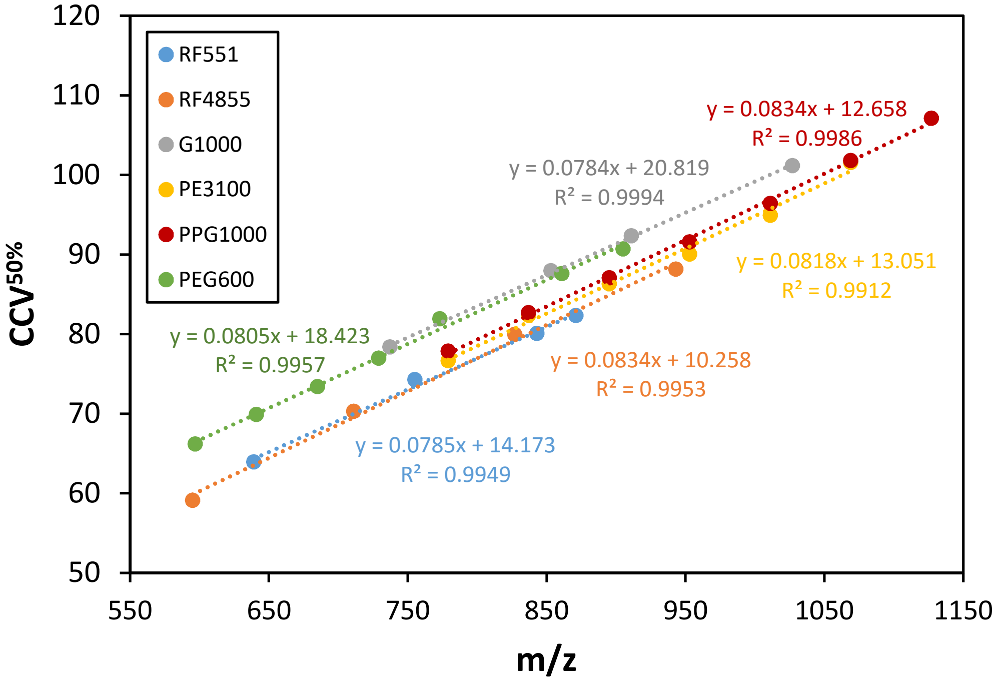

Figure S3. CCV50% *vs m*/*z* plots of the six polyether polyols.

Publisher's Note: MDPI stays neutral with regard to jurisdictional claims in published maps and institutional affiliations.

© 2020 by the authors. Submitted for possible open access publication under the terms and conditions of the Creative Commons Attribution (CC BY) license (http://creativecommons.org/licenses/by/4.0/).
